# Factors shaping the HIV-competence of two primary schools in rural Zimbabwe

**DOI:** 10.1016/j.ijedudev.2014.05.007

**Published:** 2015-03-01

**Authors:** Catherine Campbell, Louise Andersen, Alice Mutsikiwa, Erica Pufall, Morten Skovdal, Claudius Madanhire, Connie Nyamukapa, Simon Gregson

**Affiliations:** a Department of Social Psychology, The London School of Economics and Political Science, United Kingdom; b Biomedical Research and Training Institute, Zimbabwe; c School of Applied Human Sciences, University of KwaZulu Natal, South Africa; d Department of Public Health, University of Copenhagen, Denmark; e Department of Infectious Disease Epidemiology, Imperial College School of Public Health, United Kingdom

**Keywords:** International education, Development, Educational policy, HIV/AIDS, Social protection, Zimbabwe

## Abstract

We present multi-method case studies of two Zimbabwean primary schools – one rural and one small-town. The rural school scored higher than the small-town school on measures of child well-being and school attendance by HIV-affected children. The small-town school had superior facilities, more teachers with higher morale, more specialist HIV/AIDS activities, and an explicit religious ethos. The relatively impoverished rural school was located in a more cohesive community with a more critically conscious, dynamic and networking headmaster. The current emphasis on HIV/AIDS-related teacher training and specialist school-based activities should be supplemented with greater attention to impacts of school leadership and the nature of the school-community interface on the HIV-competence of schools.

## 1. Introduction

School attendance often has positive impacts on the well-being of HIV-affected and HIV-vulnerable children in sub-Saharan Africa (Gregson et al., 2004; Nyamukapa et al., 2010). In the context of the growing emphasis on the need for schools to go ‘beyond education’, international policy accords schools and teachers a central role in the care and protection of such children, particularly in relation to facilitating their school access and their health and well-being (UNESCO, 2008, 2012; UNICEF, 2013). However, much remains to be learned about (i) the readiness and ability of schools to take on these roles, and (ii) the impacts of wider contextual factors on school efforts (Ansell, 2008).

This paper explores these issues through a multi-method study of two primary schools in a rural Zimbabwean province, one in a rural area and one in a small town. The rural school is located in a relatively settled rural farming settlement, and small-town primary school is located in a small roadside town. Compared to the small-town school, the rural school is associated with (i) higher levels of school attendance by HIV-affected children in its catchment area; and (ii) higher well-being scores among HIV-affected children. We use the method of dichotomous case comparison (Schensul et al., 1999), involving comparisons of very different cases, to flag up factors facilitating or hindering each school in providing support and care for HIV-affected children.

In Zimbabwe in 2012, an estimated 15% of adults and 2.5% of children under 14 were HIV positive (UNAIDS, 2012), and 17% of children under 14 had lost one or both parents to the HIV epidemic (UNICEF, 2012). Many school learners are affected through having to care for sick or dying parents, being HIV-infected themselves or being orphaned, and taken in by varyingly supportive relatives or carers (Robson et al., 2006; Nyamukapa et al., 2008). In contexts where the ability of adults to play their traditional role in the care, support and socialisation of children is much reduced, there is growing attention to the potential for schools to take on some of these roles (Ansell, 2008).

In recent years, schools in Zimbabwe have been severely disrupted by political and economic challenges and the retreat of many NGOs (Shizha and Kariwo, 2011). In 2008, at the height of the economic crisis, many schools closed altogether; although this situation has improved with schools reopening after the government abandoned the local currency in favour of the American dollar. However, school attendance is often conditional on the payment of school fees, particularly difficult for families living in poverty, especially in rural areas, where unemployment is high, and subsistence agriculture is challenging in times of drought. Whilst the Zimbabwean governmental social protection programme Basic Education Assistance Module (BEAM) and external interventions from international funders have played a role in providing financial support securing school fees for vulnerable children, these sources have been greatly challenged by patchy international donor support, in the context of wider social instability.

There is an urgent need for research investigating the potential for schools to support HIV-affected children in Zimbabwe (Ansell, 2008). Historically, research into African schools has tended to focus on their traditional roles in preparing children for the job market through book learning, with much less research into their role in the promoting more general child well-being (Foster et al., 2012). However the HIV/AIDS epidemic has led to a shift in this trend, and, in countries such as South Africa, Namibia, and Kenya, schools and teachers have been found able to provide children with effective support in relation to social protection (Nordveit, 2010) and pastoral care (Ogina, 2008, 2010). Conversely in Zimbabwe, researchers have argued that schools and teachers are already overwhelmed by their traditional roles of academic learning in under-resourced schools, in contexts of wider political and economic instability and low salaries (Kendall and O’Gara, 2007; Shizha and Kariwo, 2011). Many Zimbabwean teachers are said to be themselves battling with poverty and/or HIV in their own lives and are unable to solve their own personal problems let alone support or counsel students (Machawira and Pillay, 2009). Furthermore, some aspects of children’s experiences at school may actually make their lives worse. These include stigmatisation by peers (Parsons, 2012), and emotional and sexual abuse (Shumba, 2002) by teachers.

As we have argued elsewhere (Campbell et al., 2013a), much of the existing empirical research into school support for HIV-affected children is descriptive in nature. There is a need for conceptual development to support systematic attention to the pathways through which schools might support or hinder the well-being of children in their care. Our conceptualisation of ‘HIV competent schools’, outlined below, provides one possible starting point here. Furthermore much research on schools focuses on specific groups (particularly teachers or learners) with less attention to the wider community contexts in which schools are located. Studies frequently focus on teachers and on a growing battery of training programmes equipping them to provide better care and support the HIV-affected in their schools. Such papers often report on the process or outcome of health or welfare interventions initiated and supported by NGOs [e.g. training teachers in skills such as resilience promotion (Ebersoehn and Ferreira, 2011), grief and bereavement counselling (Chitiyo et al., 2008), or football based health promotion (Fuller et al., 2011)]. We throw our net more widely, through a holistic multi-method study that conceptualises schools as spaces of engagement between children, teachers, guardians and local community. Rather than focusing on externally driven interventions or training programmes, we explore the accounts these different groups give of everyday life in schools, paying particular attention to the way within-school relationships are framed by the wider school-community interface.

How do the different and multi-layered interactions amongst teachers, learners, guardians and other community members facilitate or hinder HIV competence in schools? We define an ‘HIV competent community’ as a context in which community members work together to provide optimal protection and support to those affected by HIV (Nhamo et al., 2010; Campbell et al., 2012). Such a community is characterised by a number of psycho-social dimensions. Drawing on the work of Freire (1973), we argue that an HIV competent community is a context that provides opportunities for its members to engage in *dialogue* about the problems facing the HIV-affected, and critical thinking about the obstacles to tackling these problems, and ways to overcome these (Vaughan, 2010). Community members are united in *solidarity* by a sense of commitment to working together to address such challenges. They share a sense of *responsibility* for doing so, backed up by *confidence* that they have the individual and collective strength to tackle them (Haslam et al., 2009; Sliep and Meyer-Weitz, 2003). Finally such a community ideally has strong *external relationships* with outside support, welfare and NGO agencies that are able to assist in accessing social and economic resources for responding to the challenges of HIV/AIDS (Cornish et al., 2010).

In exploring determinants of HIV competence in each of our two different primary schools, we focus on both school and context (Campbell and MacPhail, 2002), through attention to two dimensions: (i) characteristics of the school and its response to the needs of HIV-affected children; and (ii) characteristics of the community surrounding each school, the local community response to HIV-affected children, and the quality of the school-community interface. Our data analysis will flag up four dimensions of schools-related ‘HIV competence’ in the rural Zimbabwean setting.

This study received ethical approval from the London School of Economics and from Medical Research Council of Zimbabwe (MRCZ/A/1661). Our multi-method project was located within a wider study of HIV/AIDS and community resources in the region, and this paper’s authors include the demographers (EP, CN, SG), and the social scientists are (CC, LA, AM, MS, CM) who produced the qualitative findings. The demographers’ work provides a contextual backdrop for the social psychologists’ case studies with the latter constituting the central focus of the paper.

## 2. Quantitative study

Our quantitative data were taken from the Manicaland household and general population cohort survey (Manicaland Survey: www.manicalandhivproject.org), and linked information on school characteristics from a parallel survey of local schools. In the Manicaland Survey, children of primary school-going age (6-12 years) were interviewed in a random sample of 1/6 households.

A child was deemed to be attending school regularly if she/he had attended on at least 80% of the last 20 school days. Individual child well-being was calculated using an objective micro-level index based on existing indices of wellbeing. Domains included health behaviours, physical health, risk and safety and psychological health.

For the comparison of local community characteristics, socioeconomic status (SES), unemployment levels, HIV prevalence (in adults aged 15–54 years), and local community group participation were examined. SES was measured using an index of sellable and non-sellable household assets. Community group participation was defined in terms of respondents who were members of community groups that they felt functioned well, which has been shown to significantly reduce the risk of HIV infection for women and increase uptake of HIV services, general awareness of HIV, and acceptance for people who are affected by HIV/AIDS (Gregson et al., 2013). Significant differences were assessed using *t*-tests for proportions or means.

### Findings

Defining a school’s ‘success’ in terms of levels of attendance and well-being of HIV-affected children, our demographers’ findings (Appendix A) yielded a complex range of information about our ‘more successful’ rural school and ‘less successful’ small-town school and their wider contexts.

Focusing first on features of the school, compared to the small-town school, our rural school had three key disadvantages: (i) fewer facilities (no phone, power-line or piped water); (ii) a higher pupil-teacher ratio with teachers paid less than their small-town counterparts; and (iii) fewer formal HIV-related activities (no AIDS policy, no teachers with AIDS training, no after school AIDS club, no AIDS peer education used in teaching, and not admitting any child unable to pay school fees). By contrast, the urban school had a phone, a power line and piped water. It had a lower pupil-teacher ratio, with more highly paid teachers; and more HIV-related activities. The latter included an AIDS policy (covering bullying, inclusion of children unable to pay fees, and a code of conduct for children and staff). The school was fully inclusive of non-fee-paying children. One quarter of all teachers had received HIV/AIDS training, there was an after-school AIDS club, and instances of teachers using peer education in their HIV-related teaching.

Turning from features of the school to features of the communities surrounding the school, rural residents were significantly poorer than their counterparts. However the rural area had some advantages: levels of HIV were lower and there were higher levels of involvement in women’s groups, AIDS groups and burial societies. All three groups have been found to be associated with high levels of HIV-avoidance in an earlier study of HIV and group membership in the region (Campbell et al., 2002; Gregson et al., 2011).

In summary, focusing only on features of the school, the demographic findings would suggest that the small-town school would be a more promising arena for the inclusion and well-being of HIV-affected pupils than the rural one – with better facilities, more and higher paid teachers, and a series of HIV/AIDS-related school activities. However, in relation to wider community contexts, the rural school would seem a more promising environment than the small town one, with lower levels of HIV/AIDS and higher memberships of HIV-avoidant group memberships. We turn to explore our qualitative findings about school and community contexts.

## 3. Qualitative study

Social scientists conducted multi-method qualitative case studies of the rural and small-town schools, each involving indepth interviews and focus group discussions with HIV-affected children, teachers, NGO workers and local community members; draw-and-write exercises with children; and brief ethnographic observations of school settings (see Appendix B for details). Aside from a specification that one school should be rural and another urban, schools were selected on the basis of convenience.

This large and complex data set was coded in three stages. Stage 1 provided an initial summary of the multi-method qualitative material from each school using Flick’s (2009) ‘summarising content analysis’. This involved descriptive coding of findings into basic themes such as sources of support within school, teacher responses to HIV-affected children and community initiatives for support. Stage 2 clustered relevant basic themes in terms of various dimensions of HIV competence. In Stage 3 the resulting spreadsheet was trawled for evidence for the differences and similarities in how schools (see Appendix C) and members of their surrounding communities (see Appendix D) understood and responded to the challenges of supporting HIV-affected children.

Below we draw selectively on these findings to showcase similarities and differences between the rural and small-town school responses. We cluster our findings around the following headings, which highlight aspects of what might constitute an ‘HIV competent school’ in the rural Zimbabwean context: the nature of HIV/AIDS impacts and sources of support in schools; factors facilitating or hindering teacher confidence, responsibility and commitment to supporting HIV-affected learners, opportunities for peer or pupil-teacher dialogue about HIV-related challenges; the interface between school and wider community.

### Findings

#### 3.1. Nature of school-related impacts of HIV/AIDS and sources of support in schools

In our qualitative case studies, there were strong similarities in people’s accounts of the challenges faced by HIV-affected children in both schools. These clustered around two overall themes. First, how HIV-affected children’s home challenges compromised their safety and well-being due to their lack of access to basic material needs (food, clothes, and health care), heavy responsibilities at home, lack of adult care and support in households, and children’s vulnerability to abuse. These were said to have severe consequences for the physical and emotional well-being of children. Second, how children’s home challenges manifested in school, impacting their school attendance and academic performance, and making them particularly vulnerable to bullying, social exclusion and stigma.

There were also differences between the schools. At the material level, the more traditional rural area had closer family ties. Frequent references were made to rural children suffering the ill-effects of inheritance theft – where extended family of a child’s dead parent would swoop into a bereaved household and remove assets, often leaving children, or remaining household members, in even more extreme poverty. In the small town, a more transient setting with weaker family ties, HIV-affected children’s financial problems derived from different sources, particularly their struggles to pay rent, an urban requirement only. Also, extended families in the urban area were more likely to ‘chase away’ orphaned children, not the case in the rural area.

During interviews, there was also a noticeable difference in the way HIV-affected children in the rural and small-town schools communicated about their life worlds. Small-town children spoke more openly about their everyday life challenges than rural children. They were also more visibly distressed, with several crying during interviews.

Informants from both settings referred to three sources of support that HIV-affected children received from schools. The first was support from teachers, most commonly material support including donations of books, uniforms or food. Some teachers kept social records of children’s home situations and in both schools teachers tended to play a role in identifying and referring children in need to external sources of support. The second source of support within the school came from peers, including offering emotional support, sharing food and school materials or assisting friends with home chores. Thirdly, learners in both schools were said to benefit from the high symbolic currency of education. With strong value attached to schooling, and references to HIV-affected children who performed well in class despite their life challenges, garnering respect from their peers and teachers in the process, school attendance was a route to positive identity in both schools.

**Fig. 1:**
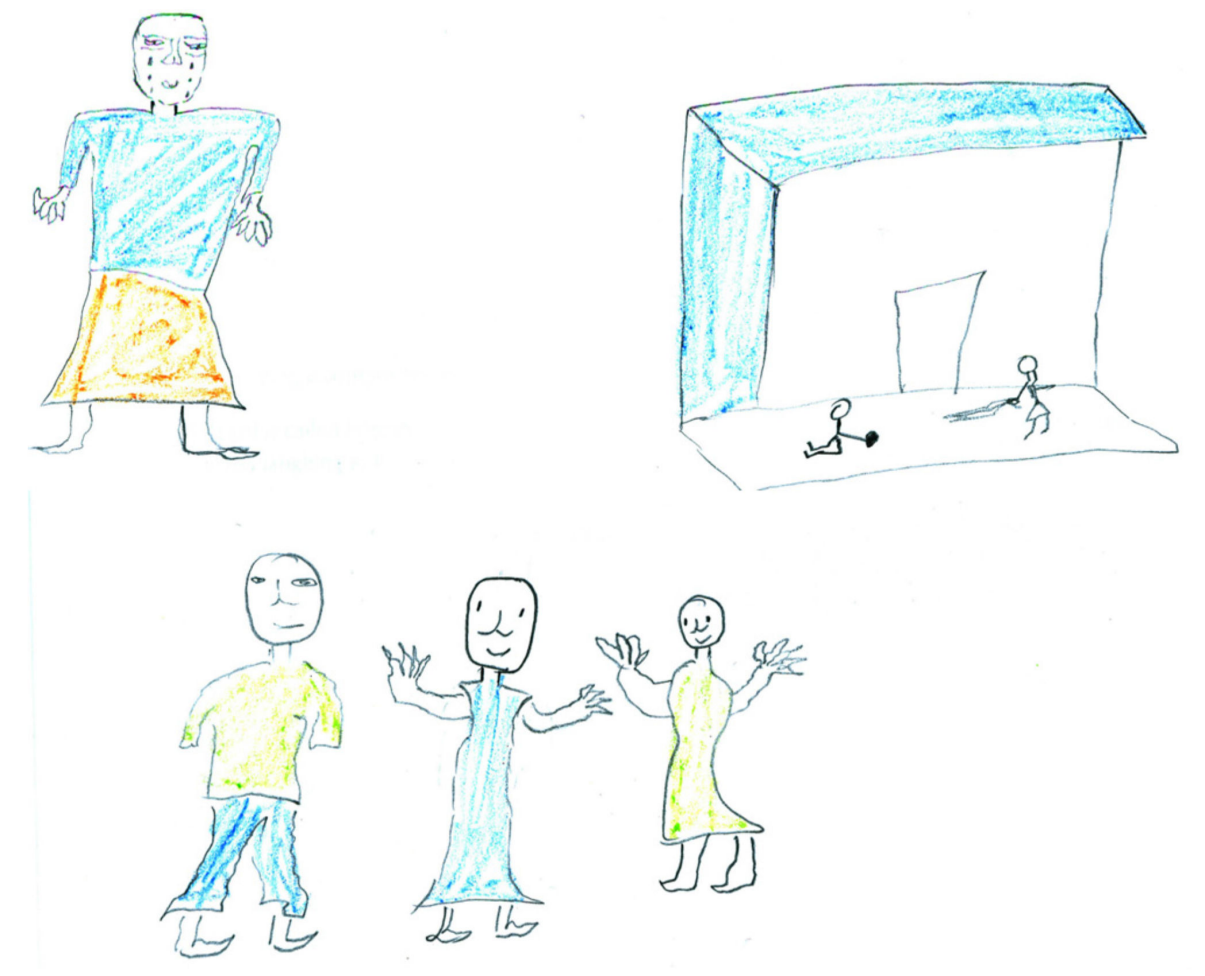
Draw and write about an HIV-affected child at your school (single child produced two drawings and captions). Top picture: *The girl’s parents are all infected with HIV. When people knew the problem they started laughing at her. At school she was so lonely and no one went near her – saying ‘if your parents have HIV you have it too’. Sometimes she spends most of her time in tears* … Bottom picture: *We bought some blankets for her parents and we comforted her. Sometimes we went to their house and help her wash her parents and their clothes. After that everyone played with her and she became very happy again*. (Rural school learner)

**Fig. 2:**
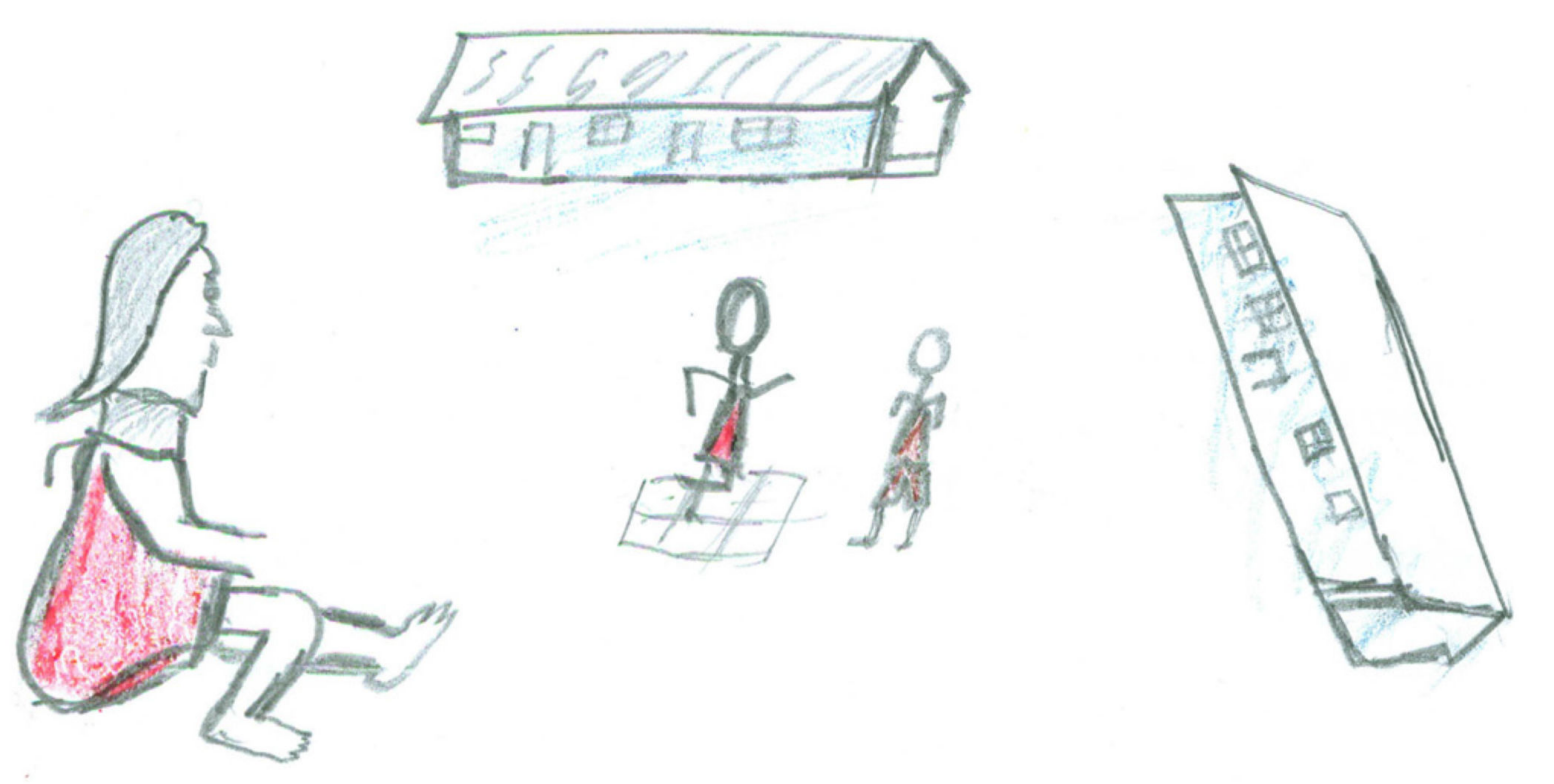
Draw-and-write. *She is sitting while the others are playing. I feel sorry for Mona because the other girls don’t want to play with her because they say that she has HIV*. (Rural school learner)

However, as will be discussed below, teacher and peer support was not widely available or offered in any systematic way in either setting. Teacher support tended to originate from kind individuals rather than from institutionalised school practices or policies. In both settings teachers varied widely in their dedication and initiative, as well as their interest in and awareness of children’s home situations. Peers were as often referred to as agents of persecution as of support. On the one hand, children often spoke positively of the support HIV-affected children received from peers (Fig. 1). But they spoke just as frequently of emotional and physical abuse of HIV-affected children by their peers through bullying and beatings or through more subtle processes of exclusion and stigma (see Fig. 2).

#### 3.2. Factors facilitating or hindering teacher confidence, responsibility and commitment for supporting learners

Across both schools teachers expressed strong pride in their role as teachers, and in the general quality and reputations of their schools. They also gave often eloquent accounts of the roles schools should play in supporting HIV-affected children. They showed a deep understanding of the potential for schools to serve as protective and inclusive environments ensuring the well-being of all children, and for teachers to take on roles beyond academic duties.

A school should be like a home. Teachers should be like parents and there should be love. The school should have an environment where a child is happy to be. You should be able to know the background of each child even if you have forty five pupils. They are yours. You are the parent. The first thing is that a teacher has to love. If you have love, it means that you will seek to understand the background of the child which you are teaching and see how best you can assist that child. (Small-town teacher)

As a teacher you are supposed to find the reason why the child skipped school. Then try to understand it because the child’s problems may be many and we have cases of some children who skip school without the knowledge of the parents. The child may have experienced some problems. (Rural teacher)

However, idealised accounts of their potential roles were rarely matched in practice in either school. Deeper attention to interviews with teachers, as well as the accounts of children themselves and community members, suggested that, in reality, teachers lacked the time, resources and motivation to offer this support. Many had little or no knowledge of the home situations of children, or of the wider community or NGO support to which they might have referred children in need. Neither school had adequate funding or resources to support children’s non-academic needs.

There were some notable differences between the rural and small-town schools however. On the positive side, the rural headmaster showed a much more critical understanding of, and willingness to reflect on, the limitations in his school’s response to HIV-affected pupils, openly acknowledging the multiple ways in which they fell short of the ideal. He also made more frequent references to HIV-affected pupils’ emotional needs than his small-town counterpart. He also appeared to have a much deeper critical understanding of the wider social factors shaping children’s problems and teachers’ responses. He engaged very deeply in the interview, with very obvious angst, and approached the researchers some days after the interview with a written document in which he elaborated further on the nature of the problems faced by his school, and on the reasons why their AIDS response was so lacking:

I wish to argue that poverty is a major contributor … It affects everyone’s morals, health and participation in school work. Teachers’ morale is also affected. Teachers’ status in the community is affected, as they appear to be depending on parents’ gifts/incentives … . Almost all schools closed in 2008 … At no other time had we experienced the same hardships. Since then, conditions of service have been poor. The teachers’ morale and commitment is very low. Moonlighting is common among teachers. The headmaster’s legitimate power over teachers is affected because his reports have no rewards in such an economy. Generally, trust is reduced because of the hardships. When school-based health workshops are offered, adoption and implementation is slow in difficult times. The influence of the teacher is comparatively lower when the job has a lower dignity. (Letter from Rural Headmaster to Researchers).

Relative to the small-town school, it appeared that the rural headmaster had been much more active in liaising and getting, albeit small-scale and temporary, one-off instances of project support from various NGOs for various school needs, and that he took a holistic approach that often went beyond his formal academic role in conceptualising the need and seeking out assistance in meeting it. Thus, for example, at the time of our interviews, he was energetically involved in trying to improve the rural school’s dismal exam performance. Acting on his view that the problem was exacerbated by the fact that many children in his school had nowhere to study on weekends, and that poor nourishment was a source of poor academic performance, he had made it obligatory for older children to attend homework sessions at the school on Saturday and Sundays, and had persuaded an NGO to donate sacks of grain to the school so that he was able to feed children attending these weekend sessions.

In contrast, the small-town headmaster gave an unmitigatedly positive account of his school. As evident from the demographers’ findings, his school had indeed been successful in putting an HIV/AIDS policy into place, which was not the case in the rural school. Furthermore, he spoke of initiatives he had set up including a small school-based poultry project to raise money to help learners who were unable to pay school fees, and to teach children farming skills, and of implementing abstinence-based sex education for pupils in years 4 to 7. His interview suggested that he had put work in setting all this up, so in many ways his relatively positive account of his school would appear to be justified. However compared to his rural counterpart, he was less inclined to engage in any sort ofcritical discussion of the limitations of his school’s response or of outstanding challenges that the school was currently grappling with, or to ‘think outside of the box’ in the way the rural headmaster had done in setting up his weekend feeding and study groups, for example.

In the interview, the small-town headmaster’s chief concern seemed to be to create a good impression of his school, sometimes in ways that were not always corroborated by local community members. Thus for example he spoke glowingly of his school’s “very effective” teacher–parent communication. This view was contradicted by parents in a community focus group who spoke of the school as a “non-starter” in relation to teacher–parent communication:

There is no collaboration with the school, our problem is that the headmaster and the parents do not see each other … we do not understand each other. So if you are talking about help from the school for children with problems, there is no such help. The school is a non-starter. (Small-town community focus group)

Compared to the small-town school, the rural school was associated with many more references to morally bad teachers, varying described as drunk at work, absent or liable to administer harsh physical punishment to children.

Some teachers even come to school drunk. Sometimes one might can come drunk for a week, not coming for lessons. Another may be drunk and come for lessons but be very harsh towards the children, even if they have not done anything wrong, the teacher may beat them. (Rural focus group with HIV-affected pupils)

Rural teachers repeatedly spoke of their low salaries (not the case with small-town teachers), preventing them from solving many of their own personal and family problems.

The bad part of teaching is that the wages are poor, so you cannot become someone who is prosperous, this destroys the motivation of teachers. If teachers are not paid well, they come to school and stay in the staff room, they will be on permanent ‘go slow’. (Focus group with rural teachers)

Rural teachers repeatedly referred to their poor salaries as an affront to their own self-respect and also to their dignity and standing in the community. Reference was made to a joke community members were fond of telling, in which a schoolteacher was found to be stealing money from a pupil, ‘because the child’s pocket money was higher than her salary’. Furthermore, their low salaries were said to make teachers more dependent on under-the-table cash ‘incentives’ from parents, making them more likely to punish children whose families failed to pay incentives.

The problem that I am seeing in schools there are some teachers that no longer have the passion to teach children. Teachers want money in order for them to teach. The orphans do not have money so they are sent away. The teachers’ priority is to get incentives from parents. (Focus group with rural teachers)

Fieldworkers observed that, in one rural school class, children whose parents had not paid incentives were made to sit at the back of the class, with their backs to the teacher and the blackboard.

By contrast, better paid and hence less demoralised teachers, and a strong and explicit Catholic ethos, appeared to be linked to higher professional standards amongst teachers in the small-town school. No references were made to teacher drunkenness at all, with fewer references to teachers beating children, and more frequent references to teachers’ dedication to caring for children. The only criticism of the ethics of the teachers came from community members who spoke of a tendency for teachers to favour the advancement of their own children, with more references also being made to general pupil favouritism amongst the teachers.

A teacher’s child and a poor man’s child should sit together. What we see is not right, that poor children sit in threes on benches at school. Yet the teachers’ kids and kids from successful families sit one to a bench, with the (favoured) kids being seated close to each other. (Small-town community, focus group)

#### 3.3. Opportunities for peer or pupil–teacher dialogue about HIV-related challenges

Dialogue has repeatedly been identified as a key medium enabling effective HIV prevention, AIDS care and support (Campbell et al., 2013b). To what extent did each school support dialogue about HIV/AIDS-related challenges? In both schools there were indeed some opportunities for dialogue between HIV-affected children, teachers and peers, but obstacles often challenged these.

Whilst both headmasters expressed a commitment to providing school-based HIV prevention education, and it did indeed appear that some was being done in the small-town school, there were huge obstacles in both settings. Teachers spoke of having insufficient knowledge, time and materials to discuss HIV or its associated challenges with learners in their care. Although both headmasters said their teachers had been trained in HIV education and that all children in the older classes had received it, in interviews, many children and teachers in both schools were not aware they had received any HIV education or training, and almost all believed their knowledge was inadequate.

Q: Are there any teachers who have received AIDS training? A: I doubt that very much. I don’t think there is anyone who has received training on that. (Small-town teacher)

Q: What HIV/AIDS information and messages do you receive from your school? A: We have never been taught about HIV. Q: Do your teachers talk about sex and pregnancy? A: They don’t talk about that. (HIV-affected rural school pupil)

In relation to care and support of HIV-affected children, both schools had policies requiring teachers to conduct home visits of children who were repeatedly absent, or exceptionally sick at school. Nevertheless, these were said to happen seldom, if at all. In the rural area, however, it was clear that teachers had far more informal contact with children and their families whilst going about their daily lives. The rural teachers clearly knew more about particular children and their home problems than was the case amongst small-town teachers. It appeared that, in the more highly populated urban area, teachers were far less likely to have informal encounters with children or their parents out of school and hence to learn about children’s personal problems in the course of their everyday lives.

At the level of informal interactions, peer support of HIV-affected children appeared to be a positive feature of both schools, allowing children to discuss their experiences and to mobilise emotional support and practical advice from fellow pupils. However, interactions between HIV-affected children and teachers – where HIV-affected children had the opportunity to share their concerns, and teachers gave them support or advice, or referred them on – were rare.

A number of reasons were given for this. As already mentioned, teachers, especially in the schools, often had little knowledge or understanding of the children’s home challenges. They said that their ability to engage with children was highly limited by high levels of HIV stigma, and the unwillingness of the vast majority of HIV-affected children (and indeed also HIV positive teachers) to disclose that they or their families were HIV positive. This made it challenging for teachers to single out children – whose plight might be very obvious in various ways – for fear of causing offence to the child or his or her family, and exacerbating the child’s sense of inferiority.

It is something that is difficult. This disease is complicated. Sometimes it’s clear that a child is HIV-affected, but it would be impossible to approach him or her or treat them differently. Special treatment would make them feel inferior. They would feel that you were stigmatising them. (Rural teacher)

Teachers emphasised that for the dignity and respect of a child he or she should be treated as equal to the others, and that singling out particular children would lower the child’s status in the school, and exacerbate their sense of inferiority, or be taken amiss by parents.

You see we might have something to donate – like old clothes and sometimes you give those things to a child. At times the parents won’t accept that so it’s tricky. They just feel ‘you are too much into our affairs’ they just don’t want that. (Rural teacher)

Another factor limiting pupil–teacher engagement was the rule in both schools that teachers were only allowed to speak English to pupils. Many children had virtually no English at all, let alone the ability to discuss extremely sensitive and painful personal issues which would require a degree of linguistic skill. Children did not see any opportunity to approach teachers on personal issues. In the rural school, teacher–pupil relations were referred to as authoritarian and hierarchical – with often no context for the child to approach a teacher off his or her own initiative, as explained by the head master:

When I took up my post, I was appalled by fear prevailed in the school. Elderly people in the community often praised the discipline in the school before 2008 (school closures due to national economic meltdown). This discipline was actually military order where pupils had no chance to discuss issues with adults. This made pupils docile and made it unlikely that teachers would understand the pupils’ way of thinking. The impression I got then was of pupils who ignore me, avoid me etc. In 2008, reports of pupils being beaten up at home and in class were very common. In a parents meeting in 2009, parents complained that pupils were not controlled in a manner they used to know (i.e. through corporal punishment). The consequence of that type of control was fear by children. Pupils could not report abuses in such state of fear. For example, the (sexual) abuse of a grade 6 pupil was only realised when the pupil was absenting from school. (Rural Headmaster)

Furthermore, teachers from the rural community frequently said that their own problems hindered them from engaging with children’s problems. These attitudes were often said to be a barrier preventing children from approaching teachers:

I can say that usually a child might not be aware of which channel to use in order to get assistance on the challenge they will be facing. Also, a child might come to me whilst I have my own social problems. So when the child approaches me, he might find me in a bad mood, and will then be scared away. (Small-town Teacher)

However, in both schools, references and attitudes to teachers varied, and there were a few references to individual teachers who were kinder than others, allowing children to build a friendly relationship with teachers and sharing their concerns, especially in the school:

Some children make friends with their teachers – such that they feel comfortable to talk to their teacher about (personal) things. I have a daughter at home, who does not want to talk, she is quiet. But with her teacher at school she talks about anything. The teacher tells me how to treat her, because she has learnt she is a slow learner and needs patience. So I see that this relationship is also there with teachers. (Small-town Community focus group)

With some exceptions, however, it appeared that the more religious and urban ethos of the small-town school might have been more supportive of the notion of offering emotional support to children than the more secular and rural school. In response to specific questions about this, the small-town headmaster spoke of two teachers qualified to counsel children in emotional distress. In interviews with rural teachers, they tended to understand ‘counselling’ in terms of discipline and punishment. In turn the rural children said their fear of teachers prevented them from approaching them with their private concerns.

#### 3.4. School interface with wider community

Both communities spoke of instances of various sources of NGO and wider community support for HIV-affected children. In both communities, local CBOs had carried out several one-off programmes of support for HIV-affected children such as poultry-breeding projects to raise money for school fees and food programmes. In the rural area, there was frequent mention of one particularly effective rural CBO that appeared to have promoted good collaboration amongst community members, the health clinic and school. However, it must be noted that, in both communities, CBO support was said to have decreased markedly in recent years, and in both settings several informants said it had been more than a year since they had last observed any CBO initiatives.

We used to receive support for the children, giving them uniforms and exercise books, meals and cooking oil. Those are the things that we would get. But it’s been almost a year since we have received that kind of support. (Small-town community focus group)

In both communities, international NGOs had carried out projects supporting school fees, food programmes and school buildings. However, NGO support was described as unevenly distributed, patchy and decreasing with reduced levels of HIV/AIDS funding, particularly from international donors in the context of global austerity, cuts in development aid and changing donor fashions. Furthermore in both communities the NGO support that was offered (i) tended to focus on material supports for HIV-affected children, helping them with school fees, books, uniforms and occasionally food parcels rather than any type of emotional care or social protection; and (ii) was frequently said to focus predominantly on orphans, neglecting children with sick parents, who were often seen to be equally or even more vulnerable and in need of support. Another limitation of NGO support – mentioned only in the rural community – was the unfortunate tendency for unscrupulous community members to steal funds from local community projects.

In both the rural and small-town areas, people said there was little informal local community support of HIV-affected children.

There is nothing done by the community (to support HIV-affected children). There is not even a single thing that is being done by the community. (Small-town community focus group).

On the contrary, there were frequent references to local community members failing to adequately care for, or worse, to actively exploit HIV-affected children (in the form of sexual abuse, expecting them to engage in unduly heavy chores and so on). Various reasons were offered for the lack of local community care for HIV-affected children. The first was financial, with the general poverty of community members leaving them with few resources for their own survival, let alone for helping others.

I also have my own children that are also being chased from school. I won’t be able to help another child that is in another house who doesn’t have parents because I am failing to take my own child to school, I am also struggling. No one can be able to assist a child thatis sitting and that has lost parents. We won’t be able to do that because we have our own problems. I struggle, when I sell vegetables I save and pay to the school and plead with the teacher. If my vegetables are bought I will pay that money. There is nowhere else I can get it from because I am not employed. (Small-town community focus group)

Some adult informants in each community blamed children for adult unwillingness to offer help, saying that many children had an inappropriate and overblown sense of their own entitlement. They disapproved of children using NGO ‘rights’ discourse in the absence of equal attention to their ‘responsibilities’.

The social structures in our culture – which used to support children – are destroyed. People, even children, misunderstand this issue of rights because they want to exercise their rights without exercising their responsibilities – they end up ignoring what is expected of them in return. (Small-town community focus group)

Other community members said the unstable NGO presence had created a culture of dependency amongst some HIV-affected children, who had come to expect that various forms of support were their ‘right’. However, the fickle nature of the NGO presence meant that no effort had been built to ensure that this support was sustained once NGOs withdrew, leaving a problematic vacuum which impoverished local people were unable to fill. Some also expressed disagreement with the assumption by many NGOs that HIV-affected children were needier than others.

The final reason given for poor community support for children and schools was a general lack of communication between teachers and community members, already alluded to above. Teachers themselves regretted what they perceived as lack of parental support for their schools manifest in e.g. some parents who refused to pay fees, others who did not turn up for parent–teacher meetings and so on.

The main problem that we face is that some parents that we call in to talk do not turn up at times which is very frustrating. Although they may know that we want to talk about their child’s health, she doesn’t turn up or he doesn’t turn up at all … The teachers may be interested but the community is not interested. They may come once but they will not come for the second meeting making everything fruitless. (Small town teacher).

However, by the same token, many parents spoke of a lack of opportunities to talk to teachers, infrequent formal meetings, and no opportunities for informal access. There appeared to be little broader ethos of parent–teacher communication or cooperation to enhance child protection and pastoral care.

That relationship should be there whereby a parent can feel free to go to the school – so every parent will always know what will be happening at the school. Sometime you will just hear it from gossip. Those from the school should remember that there are certain things that you need the parents for, not only to call them on the day you want to raise the school fees. (Small-town community focus group)

In short, across all the groups in our study, there was a general sense of poor communication within schools, and between schools, the outside community and NGOs.

However, despite the fact that informants from both schools and communities spoke of lack of community support for the school, it was clear that general levels of community support and cohesion were considerably higher in the rural area than the urban one, where more frequent reference was made to external sources of support for HIV-affected children. Rural residents mentioned the existence of a Child Protection Committee, though no details were offered regarding its operations or effectiveness. They spoke of the support that local churches offered children, particularly with counselling and school fees. They spoke of community groups that offered children assistance with food and funeral expenses, and tried to protect them through keeping an eye on neighbourhood security. They also spoke of local groupings helping HIV affected households through home based care, and offering children advice on agricultural skills; and also spoke of extended family members being available to support HIV-affected children in some situations.

Apart from occasional church support, none of these positive factors were mentioned in the small-town area. On the contrary, frequent reference was made to lack of adult awareness of children’s rights which made them unlikely even to recognise, let alone report, physical or sexual abuse of children. They said that, in an unsettled urban community, there was a general ethos where care and support of children was seen solely as the responsibility of the nuclear family, and not the community. Children reported that acts of community assistance that did take place tended to be minor, and offered “unwillingly”.

There are people who help me but they are not very willing to help us. They just help us because we will be suffering but they won’t be willing to do so. If we tell them that we have a problem they first insult and scold us, then give us money, but the money won’t be able to buy what we want to buy. (Small-town HIV-affected child)

In this place, the child does not belong to the society or community (as was traditionally the case) so no one has anything to say about someone else’s child, you just leave them doing whatever they want. (Small-town community focus group).

The small-town data were full of references to prostitution and criminal activity by parents, which were less of a problem in the more staid and settled rural community. Such parents were often said to discourage school attendance and to prioritise money for alcohol over school fees.

The parents in prostitution are not doing good because if a child says I have a headache I don’t want to go to school, that child will allowed to sit at home. The parent knows that school is not important, if the child fails in school, she can also do my job. (Small-town community member)

Sometimes as parents we don’t care. Like some of us, getting drunk (everyone laughs). Beer first, we don’t prioritise the child’s education. (Small-town community member)

## 4. Discussion

Viewing schools as spaces of engagement between learners, teachers, and less often NGOs, health services and local community residents, we have provided comparative case studies of factors impacting on school responses to HIV-affected children in two very different settings. Guided by our interest in developing better understandings of what might constitute an ‘HIV-competent school’, understood in terms of the psycho-social pathways through which school-relevant actors might provide children with pastoral care or social protection, we have shown how school responses are mediated by the presence or absence of: solidarity with the HIV-affected; commitment to supporting them; opportunities for dialogue about the challenges of HIV/AIDS; and the quality of the school-community interface.

Overall, our case study findings suggest that neither school was providing much support for HIV-affected children. Yet, as reported above, the statistical analysis found the rural school to have significantly higher levels of school attendance and well-being of HIV-affected children than the rural one. Whilst our research design prevents us from making linear claims or causal connections between our case study findings and these quantitative outcomes, our case studies have thrown up a series of interesting correlations. We use these as the basis for a series of tentative claims about the way in which features of the school and context might clash or support one another in ways that promote or hinder the likelihood of HIV-competence in schools. We look at features of the schools and their surrounding communities in turn, using the concepts of bonding and bridging social capital as a frame of integrating our findings. Bonding social capital refers to solidarity within a group (in this case the school), and bridging social capital to links between a group and external networks (in this case the school and the community) (Putnam, 2000).

In relation to features of schools, as already mentioned, our demographers’ findings might have led us to expect that the small-town school was much better placed to support children. The school had an HIV/AIDS policy; various formal AIDS activities; inclusion of non-fee paying children; relatively well paid, motivated and disciplined teachers, who were less likely to deliver harsh physical punishments, and more familiar with the notion of counselling. In addition, the academic provision and educational achievements in the school were higher. In comparison, the rural school had no specialist AIDS policy or activities; absenteeism – and even drunkenness – amongst demoralised and relatively low paid teachers, many battling unsuccessfully with their own problems; and a relatively harsh and rigid approach to discipline, with children far more fearful of teachers. After ethnographic observation of both settings, Zimbabwean co-authors of this paper said they would have had no hesitation in choosing to send their own children to the small-town school rather than the rural one had they been asked to make such a choice.

Part of the explanation for the potentially surprising advantage of rural school children in relation to school inclusion and well-being may relate to differences in the features of the local communities in which the schools were located. Despite the small-town school’s many internal assets, the rural school had a more effective school-community interface and higher general levels of social cohesion and social support in the surrounding community. Levels of poverty were higher, but subsistence farming was always a possibility when all else failed, not the case in the heavily populated small town setting where land was scarce. There was less crime and prostitution and lower levels of HIV/AIDS in the rural setting. Furthermore, there were higher levels of community-level social capital. In relation to bonding social capital, there were more extended family safety nets for children in the more long-standing rural settlement. There were also higher levels of general community participation in well-functioning women’s, burial and AIDS support groups. In relation to bridging social capital, the chances of teachers and children being known to one another through informal community contact was relatively high in a small and more tightly knit setting. Thus, teachers were much more likely to have out-of-school knowledge and interactions with their pupils. Furthermore, the more socially concerned and energetic rural school head, with his greater critical understanding of and attention to the social and historical roots of the problems facing his school, had worked much harder to build school-NGO links of various sorts than his counterpart.

It may be the case that the location of the rural school in a denser mesh of informal community networks had supported children’s opportunities for higher levels of school attendance and better well-being than the school, despite the latter’s higher socio-economic levels. In a small town setting, children lacked access to the same degree of bonding social capital, with lower levels of community group participation, against a background where the nuclear family was often children’s only source of support, with fewer safety nets for children challenged by parental death or illness. In relation to bridging social capital, there were far fewer personal networks linking children and teachers. The headmaster, less likely to dwell on the social drivers of local problems, had devoted less energy to making links with external NGOs. And more widely, the community was challenged by higher levels of prostitution, AIDS and criminal activity.

The generalisability of ethnographic case studies to other contexts is best assessed on a case by case basis by skilled social observers (Flyvbjerg, 2001), and the complex interface of school and community will map out in different ways in each setting. However, our findings suggest that, whilst the currently dominant research and intervention focus on teachers and teacher training (e.g. in counselling skills to support children) is clearly vital, there may be also a pressing need for a more systematic additional focus on the school-community interface. This would include greater attention to issues such as how best to optimise informal interactions between teachers and pupils, how to strengthen school leadership to facilitate better school-community engagements – and how to develop links between schools and well-functioning local community groups. Such work would best be supported in two ways. Firstly by developing the ability of head-teachers to think critically and systematically about how learners’ life challenges are impacted by community level relations in their particular settings. Secondly by strengthening and tailoring local support networks to better recognise and respond to children’s support needs in partnership with schools.

## Appendix A. Demographers comparisons of schools and communities

**Table:**

Type of area	Rural	Small-town	*p*
Average SES	0.30 (0.29–0.32)^a^	0.35 (0.34–0.37)^a^	<0.001
Unemployment level	60.0% (55.4–64.5%)^a^	56.2% (53.1–59.4%)^a^	0.19
HIV prevalence (ages 15–54)	13.9% (10.7–17.1%)^a^	19.8% (17.3–22.4%)^a^	<0.001
Inclusion and child health in HIV-affected learners			
School attendance by OVC	100%	84%	0.03
Well-being of children who attended regularly	+0.032	−0.019	0.007
Adult participation in local community groups			
Women’s groups (females only)	27.5% (23.4–31.7%)^a^	9.8% (7.9–11.6%)^a^	<0.001
Burial societies	33.1% (28.7–37.5%)^a^	10.2% (8.3–12.1%)^a^	<0.001
AIDS groups	15.9% (12.5–19.3%)^a^	6.4% (4.8–8.0%)^a^	<0.001
School characteristics			
Student:teacher ratio	36.2 students:1 teacher	29.2 students:1 teacher	N/A
School fees (per annum)	$45	$75	N/A
Electricity	No electricity	On grid, with power cuts	N/A
Water	No piped water	Piped water	N/A
Phone line	No phone	Has phone	N/A
HIV/AIDS response			
School has an HIV/AIDS policy	No policy	Has policy	N/A
Policy covers HIV/AIDS-related bullying	N/A	Yes	N/A
Policy support for non-fee payers	N/A	Yes	N/A
Pupils sent home for non-fee payment	15/15 (100%)	0/20 (0%)	
Policy includes code of conduct for children and staff	N/A	Yes	N/A
Teachers with AIDS training	0/10 (0%)	5/19.5* (25.6%)	
After-school AIDS club	No	Yes	N/A
Peer education used in teaching	No	Yes	N/A

a Average with 95% confidence interval in brackets.

## Appendix B. Qualitative data sources

**Table:**

Rural primary school	Small-town primary school
**HIV-affected school learners:**	**HIV-affected school learners:**
Interviews: *n* = 6	Interviews: *n* = 6
**Students:**	**Students:**
Draw-and-write: *n* = 150	Draw-and-write: *n* = 150
**Teachers:**	**Teachers:**
Primary school teachers interviews: *n* = 3	Primary school teachers interviews: *n* = 3
Focus group discussions: 1 (*n* = 6)	Focus group discussions: 1 (*n* = 6)
Head master interview: *n* = 1 + letter	Head master interview: *n* = 1
**Community members**	**Community members**
Adults FGDs: 5 (*n* = 37)	Adults FGDs: 1 (*n* = 8)
Youth FGDs: 1 (*n* =7)	Youth FGDs: 1 (*n* = 8)
Secondary school teachers interview (*n* = 3)	Secondary school teachers interview (*n* = 3)
**Ethnographic observation** (2 days)	**Ethnographic observation** (2 days)

## Appendix C. Summarising content analysis: differences and similarities in the responses of rural and small-town schools

**Table:**

Research question	Practice	Similarities (representations present at both schools)	Differences	
			Rural school	Small-town school
Representations of how schools might respond to needs of HIV-affected children	Types of support	**Teachers’ support:** Material support (books, clothes, food, uniform) Academic support Awareness of home situation (home visits, social record) Flexibility for fees, parents can work at school in lieu of fees Moral guidance Refer children to external support **Peer support:** Share food and school equipment Emotional support, reduce stress **Symbolic value of schooling** School attendance as route to positive social identities	**Peer support:** Assist with home chores **Head master:** Head master has supported children directly (assisted with chores, financial support)	
	Limitations of support	Differences between individual teachers in (i) dedication and initiative to support, and (ii) awareness of children’s home situation		
Critical understandings of challenges faced by children		**Home challenges compromising safety and well-being** Lack of basic material needs (clothes, food) Child-headed household, lack of adult care Mistreated (Physical, sexual abuse) Caregiving without equipment, knowledge Poor physical and emotional health Compromised access to health care Heavy household responsibilities (chores, caregiving) **Challenges manifested in school** Life challenges impact school attendance and learning Social challenges (bullying, exclusion, stigma)	**Home challenges compromising safety and well-being** Inheritance theft	**Home challenges compromising safety and well-being** Children pay house rent Lack of extended family support, chased away from extended family
Responsibility and commitment for supporting HIV-affected learners	Confidence by key actors in their ability to respond	**Pride in role as teacher and in school** Understanding *potential* for schools as protective and inclusive environments ensuring well-being of all children	Head master reflects critically on strengths and weakness of his school	Catholic school encourages better teacher morals and dedication to care for children
	Barriers to commitment to support children	**Barriers to teacher support:** Vague commitment, limited initiative to support Vague knowledge of support available Limited knowledge of children’s home situation Lack of open communication about HIV Scant resources to meet needs of HIV-affected children Unsupportive contexts for teachers Community members believe school stigmatises HIV-affected children Limited child–teacher dialogue Children see teachers as unable to assist	Morally bad teachers (drunk, absent, beating) Teachers constrained by own problems Strong demands for ‘incentives’, punishments if children don’t pay Poor school facilities for academic progress	Teacher–child ratio too low Unequal opportunities (community believe teachers favour own children) Head master less able reflect critically
Levels of dialogue about HIV/AIDS related challenges	Opportunities for dialogue about HIV related challenges	Occasional home visits HIV education at schools Peers share experiences and advice Afternoon health clubs	Teachers live in local community, more aware of children’s home situation	
	Barriers to HIV-affected children to talk of HIV related problems	HIV education challenged (teachers insufficient knowledge, materials on HIV, limited time for health education) Genuine efforts to treat all children equal – become a barrier preventing support (extra attention cause offence, make children feel inferior) Lack of HIV disclosure (both children, their parents) Children forced to speak in English only Social exclusion, bullying and HIV stigma Children express lack of opportunities for dialogue with teachers about problems Teachers have lack of knowledge/understanding of children’s HIV-related challenges	Teacher understand counselling as discipline/punishment Children fear teachers	Due to urban location, teachers have less knowledge of children’s home situation Only resourceful children attend afternoon health clubs
A sense of solidarity within and between key groups around tackling the problem		**Solidarity between peers:** Peers = source of both support AND stigma/social exclusion **Solidarity between peers and teachers:** Lack of communication and understanding between peers and teachers	**Solidarity between peers and teachers:** Teachers feel supported by head master Good collaboration between teachers	

## Appendix D. Summarising content analysis: Differences and Similarities in Responses of Communities Surrounding Schools

**Table:**

Research question	Practice	Similarities (representations present at both communities)	Differences	
			Rural village community members	Small town community members
Representations of supportive community responses	Types of support	Individual initiatives for material support (fees, food, clothes, blankets) Moral counselling	Child protection committee (ensure children’s rights) Church support (counselling, school fees) Community groups (food assistance, funeral assistance, neighbour security) Home based care Support from extended family members Advice on agriculture	Assist children with chores
Representations of barriers to community commitment and initiatives for support		**Barriers to community support for children** Lack of community commitment to support, few references to support initiatives General subsistence challenges within community Community members struggle with own problems Lack of solidarity within community Delegate responsibility to others (NGOs, chiefs, government, etc.) Lack of communication, understanding and collaboration within community Communities not unified Children’s attitudes alienate community Financial constraints Children feel scolded, insulted and excluded by community members **Barriers to community support for school** Teachers express lack ofparental support for school (refuse to pay fees, don’t turn up for meetings) Lack of communication, understanding and collaboration between community and school	**Barriers to community support for children** Lack of open communication about HIV Lack of trust in community HIV-affected children not seen as needier than other children Irritation regarding orphans’ unwillingness to do chores for guardians	**Barriers to community support for children** Lack of empowerment or rights knowledge hinders ability to report abuse ‘Too much emphasis on rights – too little attention to responsibilities’ Lack of governmental support Social exclusion of children in community Caring seen as responsibility of the nuclear family, not the community Assist a little – but ‘unwillingly’ Community not unified, people do not help each other Community take advantage of vulnerable children **Barriers to community support for school** Prostitutes discourage school attendance by their children Parents prioritise alcohol over school expenses School does not acknowledge poverty in community Parents express lack of free dialogue with teachers Community not aware what school needs from them
Wider community context	Strengths of community	Institutionalised sources of support within community Local CBO – fees, food, encourage HIV testing BEAM (school fees) NGO support (fees, food programmes)	Well educated Access to public transport Good links between CBO, school, health clinic and community	Strong value for education Religious
	Contextual challenges in community	Challenges in community Poverty Lack of job opportunities Hunger Institutionalised sources of support NGO support unevenly distributed, patchy and decreasing Support only for fees (not books, uniforms, etc.)	Challenges in community Drought, challenged water access Few opportunities to sell farming produce Institutionalised sources of support Thieves steal from projects	Challenges in community High level of prostitution Bad behaviour, alcohol, smoking, stealing Higher HIV prevalence, people mix spread HIV Lack of job opportunities, no industry Lack of hospital access Unclean, poor toilets, many diseases Selling firewood as common source of income, challenged by too little firewood Lack of electricity Institutionalised sources of support NGO support prioritises orphans, lack of attention to children with sick parents
